# Supramolecular peptide nanotubes as artificial enzymes for catalysing ester hydrolysis†

**DOI:** 10.1039/d3py00993a

**Published:** 2023-09-14

**Authors:** Qiao Song, Zihe Cheng, Sébastien Perrier

**Affiliations:** a Department of Chemistry, University of Warwick Coventry CV4 7AL UK songq@sustech.edu.cn perrier@warwick.ac.uk; b Shenzhen Grubbs Institute, Southern University of Science and Technology Shenzhen 518055 China; c Warwick Medical School, University of Warwick Coventry CV4 7AL UK; d Faculty of Pharmacy and Pharmaceutical Sciences, Monash University Parkville VIC 3052 Australia

## Abstract

Peptide-based artificial enzymes are attracting significant interest because of their remarkable resemblance in both composition and structure to native enzymes. Herein, we report the construction of histidine-containing cyclic peptide-based supramolecular polymeric nanotubes to function as artificial enzymes for ester hydrolysis. The optimized catalyst shows a *ca*. 70-fold increase in reaction rate compared to the un-catalysed reaction when using 4-nitrophenyl acetate as a model substrate. Furthermore, the amphiphilic nature of the supramolecular catalysts enables an enhanced catalytic activity towards hydrophobic substrates. By incorporating an internal hydrophobic region within the self-assembled polymeric nanotube, we achieve a 55.4-fold acceleration in hydrolysis rate towards a more hydrophobic substrate, 4-nitrophenyl butyrate. This study introduces supramolecular peptide nanotubes as an innovative class of supramolecular scaffolds for fabricating artificial enzymes with better structural and chemical stability, catalysing not only ester hydrolysis, but also a broader spectrum of catalytic reactions.

## Abstract 2

Histidine-containing cyclic peptide-based supramolecular peptide nanotubes were constructed to function as artificial enzymes for catalysing ester hydrolysis.

## Graphical abstract

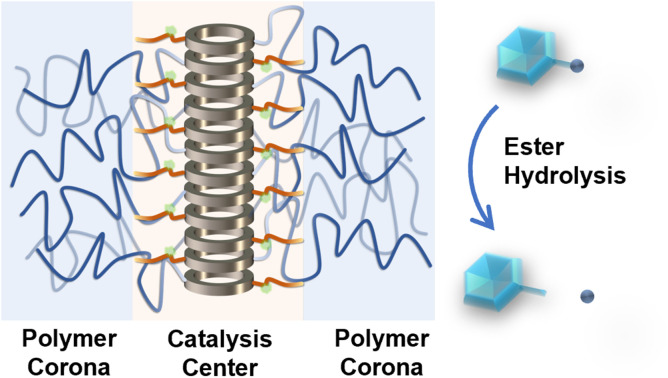

## Introduction

Natural enzymes exhibit remarkable catalytic efficiency and substrate selectivity owing to their unique catalytic microenvironment. Guided by their three-dimensional structures, the active sites are precisely positioned to maximize both efficiency and specificity. While extensive research has focused on exploring the structure–function correlations of enzymes, there has also been a concerted effort to design artificial enzymes with simplified architectures that emulate nature's principles.^1–7^ Supramolecular chemistry offers a powerful methodology for arranging catalytic components within self-assembled structures to achieve optimal performance.^8–10^ A diverse variety of ordered scaffolds, including micelles, nanofibers, nanotubes, and gel networks, have been fabricated through this approach, displaying not only comparable catalytic properties to natural enzymes but also advantages such as facile synthesis, cost-effectiveness, and improved stability.^11–13^

Hydrolytic enzymes, known as hydrolases, have received significant attention as models to mimic, due to their ubiquity in living systems and increasing industrial relevance. It has been shown that imidazolyl groups of histidine residues play an essential role in the catalytic mechanisms of various native hydrolases, such as chymotrypsin, trypsin, acetylcholine esterase, and kidney dialkylfluorophosphatase.^14–17^ To date, numerous small molecular and polymeric building blocks incorporating imidazole have been designed and assembled *via* supramolecular interactions to serve as artificial enzymes and catalyse ester hydrolysis.^18–25^ Among these, peptide-based catalytic systems attract particular interests owing to their great similarity in composition and structure to native enzymes.^26,27^ For instance, a self-assembling peptide amphiphile containing two histidine residues, reported by Guler and Stupp, assembled into nanofibers and showed significantly improved hydrolysis efficiency towards model compound 2,4-dinitrophenyl acetate.^28^ Subsequent to this pioneering work, a few more cases have been reported, employing self-assembling peptide amphiphiles or amphiphilic short peptides to catalyse ester hydrolysis.^29–36^ Nonetheless, many of these linear peptide-based assemblies are notably affected by their chemical structures, causing undesired change in their self-assembling structures when targeting specific functionalities. In addition, the potential degradation of the linear peptide motifs remains a challenge when using these artificial enzymes in biological applications. Hence, a new range of artificial enzymes with better structural and chemical stability are required.

Self-assembling cyclic peptides are able to stack into nanotubes *via* multiple hydrogen bonding interactions, including cyclic d,l-α-peptides, cyclic β-peptides, cyclic α,γ-peptides, and so on.^37–40^ The strong binding affinity contributed by the multiple hydrogen bonding enables different strategies to decorate the internal and external surfaces of these nanotubes while maintaining the self-assembling structures.^41^ Specifically, conjugating hydrophilic polymers onto cyclic peptides effectively prevents the lateral aggregation between the peptide nanotubes, and improves their solubility and stability in aqueous media, leading to the formation of well-defined supramolecular polymeric nanotubes.^42–45^ The unique nanotubular structures and the easy-modification nature of the cyclic peptides and cyclic peptide–polymer conjugates have allowed the use of such supramolecular systems in fields of ion channels,^46,47^ antimicrobials,^48,49^ drug delivery vectors,^50–52^ and optoelectronics.^53–56^ Furthermore, cyclic peptides show better stability towards enzymatic degradation than linear peptides.^57,58^ However, even though their powerful capability of molecularly arranging functional moieties along the self-assembled nanotubes has been widely recognized, their application as supramolecular scaffolds towards artificial enzyme design has been rarely explored.

**Scheme 1:**
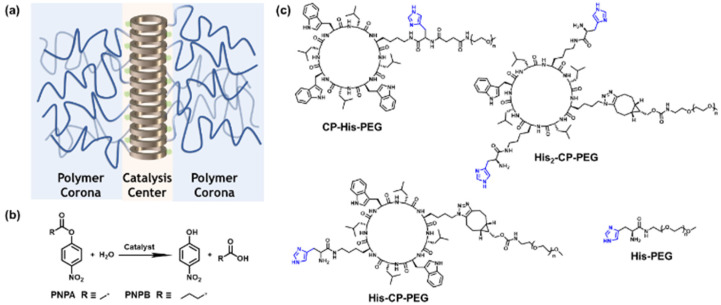
(a) Schematic illustration of the supramolecular peptide nanotube formed by histidine-containing cyclic peptide–polymer conjugate; (b) model hydrolysis reaction used in this work; (c) chemical structures of the three histidine-containing cyclic peptide–polymer conjugates investigated in this work.

In this work, we investigated the possibility of utilizing cyclic peptide based supramolecular polymeric nanotubes as artificial enzymes for ester hydrolysis. To this end, and as a proof of concept, three different cyclic peptide–polymer conjugates were designed and synthesized, with one or two histidine residues attached onto the cyclic peptide: CP–His–PEG, His–CP–PEG and His_2_–CP–PEG. Driven by the multiple hydrogen bonding interactions between the cyclic peptides, supramolecular polymeric nanotubes are expected to form in aqueous media, bringing the histidine residues together and aligning along the nanotubes, as shown in Scheme 1a. The enhanced catalytic efficacy was proved by studying the hydrolysis of a widely studied model compound 4-nitrophenyl acetate (PNPA) in PBS buffer (pH = 7.4) (Scheme 1b). The Michaelis–Menten model was used to fit the catalytic kinetics of His–CP–PEG towards PNPA, suggesting it being a real enzyme model for ester hydrolysis. Moreover, the supramolecular catalyst showed better catalytic activity towards a more hydrophobic substrate, 4-nitrophenyl butyrate (PNPB), which could be attributed to the amphiphilic property of the supramolecular polymeric nanotubes. Introducing an internal hydrophobic region within the self-assembled polymeric nanotube further promotes the hydrolytic activity towards PNPB. Therefore, the supramolecular peptide nanotubes could function as artificial enzymes towards a wider range of substrates.

## Experimental

### Materials

Fmoc-protected amino acids and coupling agents were purchased from Iris Biotech GmbH. α-Methoxy-ω-amino PEG (CH_3_O–PEG–NH_2_, *M*_n_ = 5000 g mol^−1^), α-methoxy-ω-NHS ester (CH_3_O–PEG–NHS, *M*_n_ = 5000 g mol^−1^) were purchased from Rapp Polymere. Boc–His(Trt)–OH, 4-nitrophenyl acetate (PNPA), 4-nitrophenyl butyrate (PNPB), (1*R*,8*S*,9*S*)-bicyclo[6.1.0]non-4-yn-9-ylmethyl *N*-succinimidyl carbonate (BCN–NHS) and other chemicals were purchased from Sigma-Aldrich. Solvents were purchased from several departmental suppliers, Honeywell, Fisher and Sigma-Aldrich.

### Characterization

#### Nuclear magnetic resonance spectroscopy (NMR)

^1^H NMR spectra were measured using a Bruker Avance III HD 400 MHz NMR spectrometer. The residual solvent peaks were used as internal references.

#### Gel permeation chromatography (GPC)

GPCs were measured using Agilent Infinity II MDS instrument equipped with differential refractive index (DRI), viscometry (VS), dual angle light scatter (LS) and variable wavelength UV detectors. The system was equipped with two PLgel Mixed D columns (300 × 7.5 mm) and a PLgel 5 μm guard column. The eluent is DMF with 5 mmol NH_4_ BF_4_ additive. Samples were run at 1 mL min^−1^ at 50 °C. The samples were prepared by filtering them through 0.22 μm pore size PTFE membranes before injection. Agilent EasyVial poly(methyl methacrylate) standards were used to calibrate the instrument and output data were analyzed using Agilent GPC/SEC software.

#### High-performance liquid chromatography (HPLC)

High-performance liquid chromatograms were measured using a Shimadzu Prominence HPLC, equipped with an Agilent Poroshell 120 C18 column (100 mm × 4.6 mm) with 2.7 μm micron packing. Water and acetonitrile were used as mobile phase A and B, respectively. All solvents contained 0.04 vol% TFA. The gradient used for HPLC analysis was increased from 5% to 95% B in 20 minutes. Detection was achieved *via* monitoring UV absorption at different wavelengths. Samples were dissolved in mobile phase A with concentration of 0.5 mg mL^−1^ and the injection volume was 50 μL.

#### Mass spectrometry (ESI-TOF)

ESI-TOF mass spectra were measured using an Agilent 6130B single Quad to characterize the peptides in both positive and negative ionization modes. Samples were dissolved in methanol.

#### Ultraviolet–visible (UV–vis) absorption spectroscopy

UV–vis absorption spectra were measured using an Agilent Technologies Cary 60 UV–vis spectrometer. The path length of the cuvette is 2 mm.

#### Fluorescence emission spectroscopy

Fluorescence emission spectra were measured using an Agilent Technologies Cary Eclipse Fluorescence spectrometer.

#### Small angle X-ray scattering (SAXS)

Samples were loaded into borosilicate capillaries with a 1 mm path length. SAXS profiles were measured using a Xenocs Xeuss 2.0 diffractometer equipped with a Dectris Pilatus 1 M detector positioned 5 m from the sample position with a monochromatic X-ray beam of 1.54 Å wavelength. This covers an effective scattering vector (*Q*) range of 0.004–0.15 Å^−1^ where,
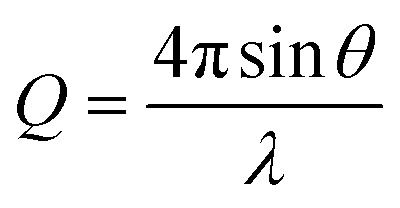
where 2*θ* represents the scattering angle and *λ* represents the wavelength of the incident photons. Data were collected for 4 h at room temperature. Data were normalized to the flux of the incident direct beam to place the intensity on absolute scaling, prior to radially averaging pixel intensities and subtraction of the background scattering profile collected from PBS buffer.

Reduced SAXS data were analyzed using model dependent analysis in SasView (https://www.sasview.org/). A model describing a core–shell cylinder was used where the core represents the cyclic peptide, and the shell represents the PEG corona.

#### Static light scattering (SLS)

The SLS data were obtained using the ALV/CGS-3 Compact Gonimeter System with a vertically polarized laser with a wavelength of 632 nm. A range of different concentrations of conjugates were measured using SLS at 25 °C. The compounds were first accurately measured in a vial and corresponding volumes of water were added to yield different concentrations. Before measuring, the solutions were filtered using 0.45 μm pore size Nylon membranes.

## Results and discussion

### Self-assembling behaviour of histidine-containing conjugates

To optimize the structure of the histidine-containing cyclic peptide–polymer conjugates, three different conjugates were designed and synthesized, namely CP–His–PEG, His–CP–PEG, and His_2_–CP–PEG, as shown in Scheme 1c. A single histidine residue is directly attached to the cyclic peptide in the case of CP–His–PEG and His–CP–PEG, while two histidine residues are attached to the cyclic peptide in conjugate His_2_–CP–PEG. The hydrophilic polymer, poly(ethylene glycol) (PEG) is linked to the histidine residue in CP–His–PEG, and to the cyclic peptide for either His–CP–PEG or His_2_–CP–PEG. To immobilize both the histidine moiety and the hydrophilic polymer onto the cyclic peptide, a chemical reaction with high efficiency and orthogonality is essential. Herein, the three conjugates were synthesized by firstly reacting histidine residues *via* amidation reaction chemistry with the amino groups on the cyclic peptides, followed by reacting either an *N*-hydroxysuccinimide (NHS) ester modified PEG (CH_3_–PEG–NHS, *M*_n_ = 5000 g mol^−1^) *via* NHS coupling or a strained alkyne modified PEG (CH_3_–PEG–BCN, *M*_n_ = 5000 g mol^−1^), with the azido groups *via* strained alkyne/azide cycloaddition, which were fully characterized by ESI-MS, HPLC, and GPC (Fig. S1–S3†). A control compound in the absence of cyclic peptide, His–PEG, was also synthesized *via* amidation reaction chemistry (Fig. S4 and S5†).

**Fig. 1:**
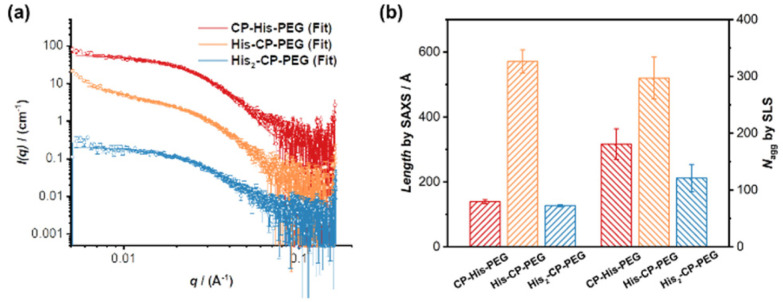
Characterization of self-assembled peptide nanotubes. (a) SAXS scattering data and fitting to a core–shell cylinder model; (b) length of the nanotubes determined by SAXS and number of aggregation (*N*_agg_) determined by SLS.

The self-assembling behaviour of the three histidine-containing conjugates in aqueous media was investigated by small angle X-ray scattering (SAXS) and static light scattering (SLS). Fig. 1a shows the scattering data for CP–His–PEG, His–CP–PEG, and His_2_–CP–PEG in PBS buffer (pH = 7.4). Using SasView software, the data could all be fitted with a core–shell cylinder model, suggesting the self-assemblies forming polymeric nanotubes in PBS buffer despite their chemical compositions, emphasizing the structural stability of the cyclic peptide-based scaffold (Fig. S8 and Table S1†). The values of the shell thickness are similar to one another due to the fact that the same polymer is used for all three conjugates. However, as shown in Fig. 1b, the length of the nanotubes (and the number of aggregation, *N*_agg_, determined by SLS, Fig. S9 and Table S2†) varies significantly. We hypothesize it is due to the physicochemical properties of different conjugate structures. The electrostatic repulsion caused by the partially charged histidine moieties at pH = 7.4 are believed to restrict the cyclic peptide–polymer conjugates from self-assembling. Therefore, inserting one histidine residue between the cyclic peptide and polymer affects assembly more than attaching it to the opposite side of the cyclic peptide–polymer conjugate, thus causing the formation of shorter nanotubes by CP–His–PEG compared to His–CP–PEG. His_2_–CP–PEG assembles into the shortest nanotubes because of the two histidine residues.

### Catalytic performance of histidine-containing conjugates

**Fig. 2:**
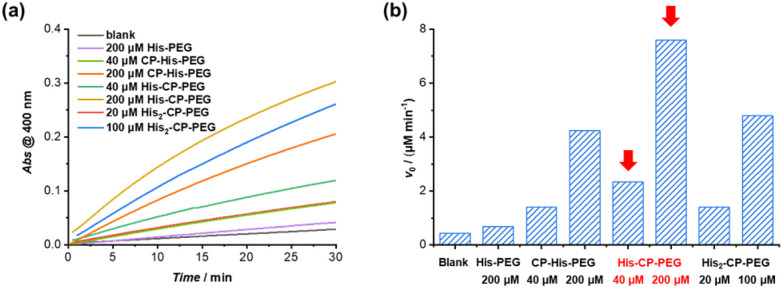
(a) Plots of absorbance at 400 nm *vs.* time for the hydrolysis of PNPA (1 mM) with the addition of different catalysts in PBS buffer (pH = 7.4); (b) catalytic reaction rates for PNPA hydrolysis (1 mM) in PBS buffer (pH = 7.4).

The hydrolysis of PNPA by the three histidine-containing conjugates was monitored by UV-vis spectroscopy at 400 nm, following the formation of 4-nitrophenol at 25 °C in PBS buffer (pH = 7.4). The imidazolyl group on histidine residue reacts directly with PNPA to form 4-nitrophenol and acylium ionimidazole complex, which in turn reacts with water to form acetic acid and the recovered imidazole group. As shown in Fig. 2, the control compound, His–PEG, at concentration 200 μM, led to a 1.6-fold increase in the hydrolysis rate of PNPA compared to the blank reaction, indicating minimal catalytic activity of non-assembling histidine groups. In contrast, the addition of CP–His–PEG, His–CP–PEG or His_2_–CP–PEG resulted in a significant enhancement of the hydrolytic rate of PNPA. At a histidine residue concentration of 200 μM, a 6.3-fold increase for CP–His–PEG, a 11.2-fold increase for His–CP–PEG, and a 7.1-fold increase for His_2_–CP–PEG were observed compared to His–PEG. Moreover, the catalytic effect was also observed at a much lower catalyst concentration (40 μM), resulting in 2.0-, 5.5- and 3.3-times enhancement for CP–His–PEG, His–CP–PEG and His_2_–CP–PEG, respectively. It is therefore suggested that the hydrolytic rate enhancement by histidine-containing conjugates arises from the presence of a high density in reactive histidine residues within the supramolecular polymeric nanotubes, forming active catalytic centres, which mimic native enzymes. The catalytic performance of His–CP–PEG is significantly superior to CP–His–PEG, which is most likely due to the larger steric hindrance caused by the adjacent PEG in CP–His–PEG. Surprisingly, increasing the number of histidine residues leads to lower catalytic efficacy, which might be ascribed to the better aggregation capability of His–CP–PEG, as evidenced by SAXS and SLS. Overall, comparing the catalytic activity of the three conjugates with different chemical structures shows that the supramolecular catalyst based on His–CP–PEG performs the best towards the hydrolysis of PNPA.

**Fig. 3:**
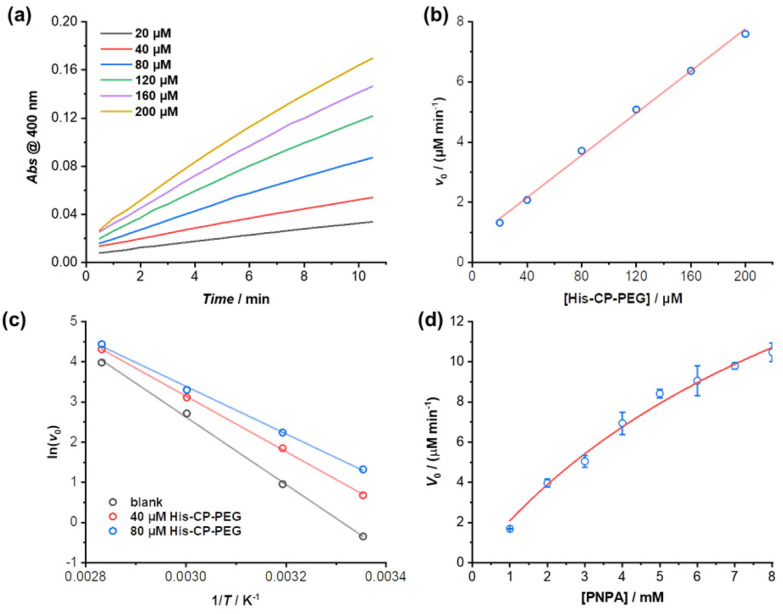
(a) Plots of absorbance at 400 nm *vs.* time for the hydrolysis of PNPA (1 mM) using different concentrations of His–CP–PEG as catalyst in PBS buffer (pH = 7.4); (b) evolution of initial catalytic reaction rates for PNPA hydrolysis (1 mM) as a function of His–CP–PEG concentration; (c) fitting of initial hydrolytic rates to reaction temperature based on Arrhenius equation to give the apparent activation energy (*E*_a_); (d) plot of initial hydrolytic rate *vs.* concentration of PNPA in the presence of 40 μM His–CP–PEG.

The enzymatic behaviour of the supramolecular polymeric nanotubes formed by His–CP–PEG was further evaluated. As shown in Fig. 3a, the hydrolysis of PNPA was significantly accelerated with the increase of His–CP–PEG concentration. The initial hydrolytic rate was found to increase linearly with His–CP–PEG concentration (Fig. 3b), which was in accordance with the reported first-order relationship between hydrolysis rate of phenyl acetates and imidazole concentration. Moreover, the linear relationship between hydrolytic rate and His–CP–PEG concentration suggests the stability of the self-assembled polymeric nanotubes at low concentration, highlighting the advantage of cyclic peptide building blocks as stable scaffolds for supramolecular catalyst design.^53^

The catalytic nature of the His–CP–PEG based supramolecular nanotubes was confirmed by a decrease of activation energy in the presence of His–CP–PEG. The hydrolysis of PNPA was monitored at different temperatures (25, 40, 60, and 80 °C) at His–CP–PEG concentrations of 0, 40, and 80 μM, respectively. Fitting the evolution of initial hydrolytic rates as a function of reaction temperatures using Arrhenius equation led to the determination of the apparent activation energy (*E*_a_) related to the PNPA hydrolysis (Fig. 3c). As shown in Table S3,† in the absence of His–CP–PEG, *E*_a_ was calculated to be 69.7 kJ mol^−1^ in PBS buffer (pH = 7.4). The addition of 40 μM His–CP–PEG led to a 12.3 kJ mol^−1^ decrease of *E*_a_ value, while a 20.5 kJ mol^−1^ reduction was observed in the presence of 80 μM His–CP–PEG. The noticeably lowered *E*_a_ observed for His–CP–PEG clearly supports the hypothesis that the imidazolyl groups on histidine residues are capable of binding with the substrate PNPA and activating the ester bonds towards hydrolysis.

The PNPA hydrolytic rates were calculated under various substrate concentrations in the presence of a constant catalyst concentration ([His–CP–PEG] = 40 μM). The Michaelis–Menten model, which describes a typical enzymatic saturation kinetics, was used to fit the data, suggesting it being a real enzyme model for ester hydrolysis (Fig. 3d). The apparent rate constant for PNPA hydrolysis with His–CP–PEG was found to be *k*_cat_ = (1.07 ± 0.15) × 10^−2^ s^−1^, while the first-order rate constant for background ester hydrolysis was *k*_uncat_ = (1.57 ± 0.01) × 10^−4^ s^−1^. Compared with the uncatalyzed reaction, this represents a 68-fold rate enhancement, suggesting that the His–CP–PEG based supramolecular polymeric nanotube could function as a very efficient artificial enzyme for catalysing ester hydrolysis. In addition, the catalytic performance of our catalyst was compared with previous reported self-assembling peptide amphiphiles and amphiphilic short peptides. As shown in Table S4,† despite that there is a broad variation in *k*_cat_, *K*_m_, or *k*_cat_/*K*_m_, our catalyst's catalytic performance falls within an intermediate range amidst these variations.

### Enhanced catalytic capacity towards hydrophobic substrates

**Fig. 4:**
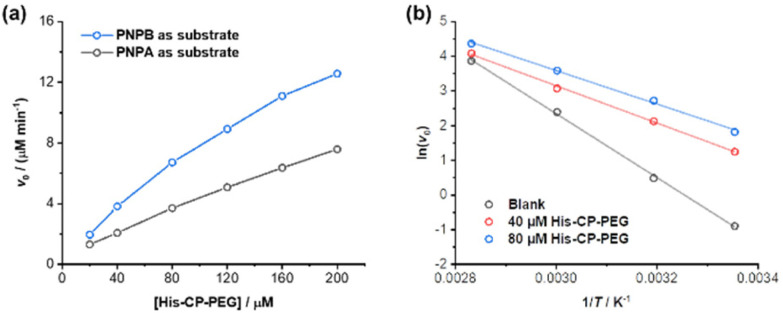
(a) Evolution of initial catalytic reaction rates for PNPB and PNPA hydrolysis (1 mM) as a function of His–CP–PEG concentration in PBS buffer (pH = 7.4); (b) fitting of initial hydrolytic rates to reaction temperatures based on Arrhenius equation to give the apparent activation energy (*E*_a_).

The amphiphilic nature of the cyclic peptide–polymer conjugates should in theory enable the supramolecular catalyst to hydrolyse more hydrophobic substrates. To test our hypothesis, 4-nitrophenyl butyrate (PNPB) was chosen as a model hydrophobic substrate. At a His–CP–PEG concentration of 40 μM, an 8.5-fold enhancement of the PNPB hydrolysis rate was observed compared to rate in the absence of any catalyst, while 200 μM His–CP–PEG resulted a 28.9-fold faster hydrolysis. Surprisingly, His–CP–PEG showed even higher hydrolysis rate when using PNPB as substrate compared to PNPA, as indicated in Fig. 4a. The accelerated hydrolysis rate could be further confirmed by the decrease of apparent *E*_a_ in the presence of His–CP–PEG (Fig. 4b). The *E*_a_ representing PNPB hydrolysis was determined to be 76.6 kJ mol^−1^ in the absence of His–CP–PEG in PBS buffer, which is higher than that of PNPA (69.7 kJ mol^−1^), implying PNPB is more difficult to undergo hydrolysis in PBS buffer due to the increased hydrophobicity. However, a significantly greater decrease (32.0 kJ mol^−1^) was witnessed in the presence of 40 μM His–CP–PEG, and the addition of 80 μM His–CP–PEG resulted in a further 4.5 kJ mol^−1^ decrease of the *E*_a_ value (Table S3†).

**Fig. 5:**
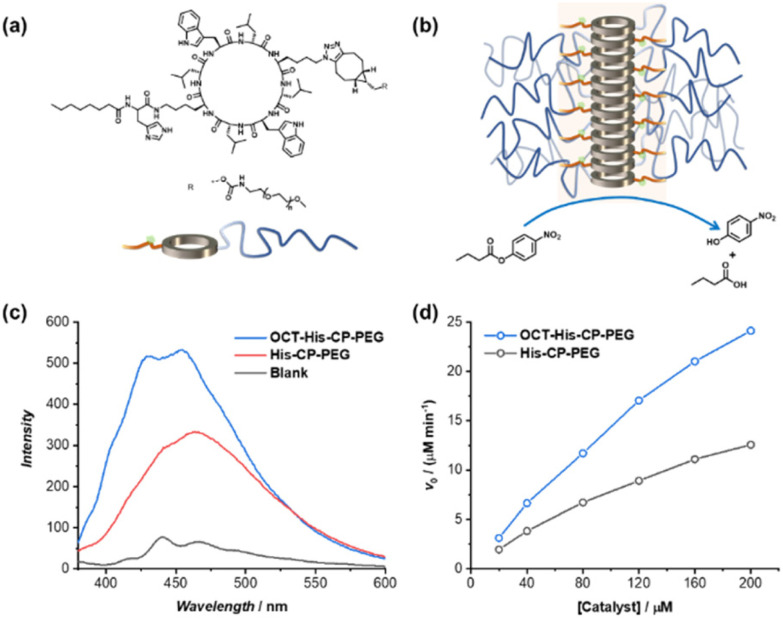
(a) Chemical structure of OCT–His–CP–PEG; (b) schematic representation showing the self-assembled polymeric nanotube with an internal hydrophobic region; (c) fluorescence spectra of DPH in the presence of OCT–His–CP–PEG or His–CP–PEG; (d) evolution of initial catalytic reaction rates for PNPB hydrolysis (1 mM) as a function of catalyst concentration in PBS buffer (pH = 7.4).

Encouraged by the enhanced catalytic capacity of the amphiphilic cyclic peptide–polymer conjugates towards hydrophobic substrates, a new histidine-containing cyclic peptide–polymer conjugate was further developed, which leads to an internal hydrophobic region within the self-assembled polymeric nanotube. Herein, *n*-octanoic acid was attached to His–CP–PEG *via* the amino group of histidine residue, resulting an amphiphilic conjugate, OCT–His–CP–PEG (Fig. 5a). As shown in Fig. 5b, the hydrophobic alkyl chains are expected to provide a low-polarity environment for the histidine residues, thus boosting the catalytic activity towards hydrophobic substrates. The formation of self-assembled polymeric nanotubes was evidenced by SAXS and SLS (Fig. S8 and Table S1†). The existence of the hydrophobic region was probed by fluorescent dye 1,6-diphenylhexatriene (DPH), which is quenched in water but shows strong fluorescence in hydrophobic environment. As shown in Fig. 5c, enhanced fluorescence was observed when DPH is mixed with OCT–His–CP–PEG in PBS buffer (pH = 7.4) compared to that of His–CP–PEG, confirming the presence of a more hydrophobic region within the self-assembled polymeric nanotube. Consequently, OCT–His–CP–PEG exhibited promoted hydrolytic activity towards PNPB (Fig. 5d). For example, in the presence of 40 μM OCT–His–CP–PEG, a 14.8-fold enhancement of the PNPB hydrolysis rate was witnessed compared to that in the absence of any catalyst, while 200 μM OCT–His–CP–PEG led to a 55.4-fold faster hydrolysis.

## Conclusions

In summary, cyclic peptide based self-assembled polymeric nanotubes have been designed to function as efficient supramolecular catalysts for ester hydrolysis. Among the three designed catalysts, His–CP–PEG demonstrated the highest effectiveness when using PNPA as the model substrate, showing an approximately 70-fold increase in reaction rate compared to the uncatalyzed reaction. Equally noteworthy is the amphiphilic nature of the supramolecular catalysts, which leads to an enhanced catalytic activity towards more hydrophobic substrates. Introducing an internal hydrophobic region within the self-assembled polymeric nanotubes, as seen in OCT–His–CP–PEG, further promotes the hydrolytic activity, leading to a 55.4-fold acceleration in hydrolysis when used in concentrations as low as 200 μM. These cyclic peptide-based supramolecular polymeric nanotubes offer a novel and versatile class of supramolecular scaffolds. They enable the fabrication of artificial enzymes with improved structural and chemical stability, facilitating not only ester hydrolysis but also a broader spectrum of catalytic reactions.

## Author contributions

The manuscript was written through contributions of all authors. All authors have given approval to the final version of the manuscript.

## Conflicts of interest

There are no conflicts to declare.

## Supplementary Material

PY-014-D3PY00993A-s001
