# Supplementary material for: Supramolecular peptide nanotubes as artificial enzymes for catalysing ester hydrolysis
Source: Polym Chem. 2023 Sep 14;14(41):4712–8. doi: 10.1039/d3py00993a (PMC10594401; doi:10.1039/d3py00993a)
Supplement: PY-014-D3PY00993A-s001 [file PY-014-D3PY00993A-s001.pdf]

Electronic Supplementary Material (ESI) for Polymer Chemistry.
This journal is © The Royal Society of Chemistry 2023

Supporting Information

Supramolecular Peptide Nanotubes as Artificial Enzymes for Catalysing Ester Hydrolysis

Qiao Song, Zihe Cheng, and Sébastien Perrier*

S1. Synthesis

1. Synthesis of cyclic peptides CP-NH_2, H_2N-CP-N_3, and $(H_2N)_2$-CP-N_3.

The three starting cyclic peptides were synthesized according to procedures reported previously.[1-3]

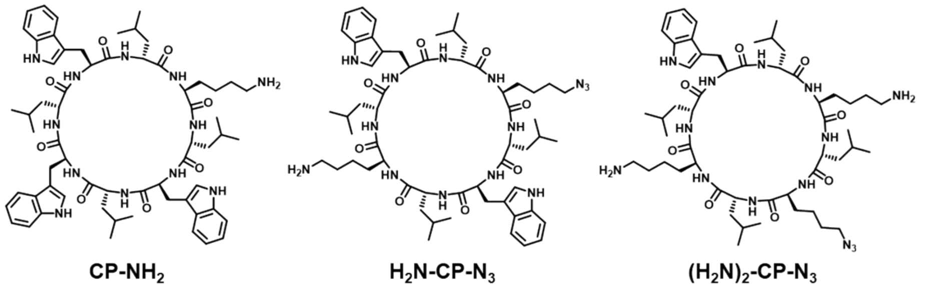

2. Synthesis of CP-His-PEG

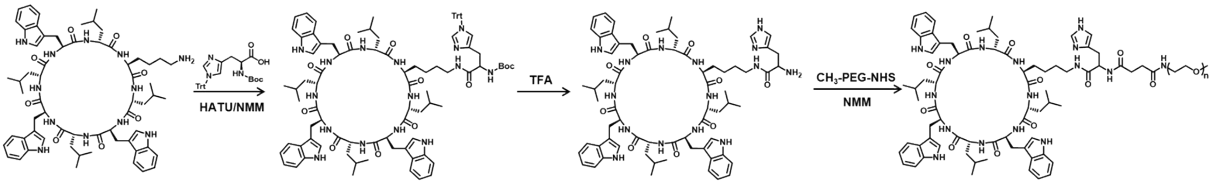

a. Synthesis of CP-His(Trt)-Boc

CP-NH_2 (50.0 mg, 0.044 mmol) and Boc-His(Trt)-OH (43.7 mg, 0.088 mmol) were dissolved in 1.5 mL anhydrous DMF, with the addition of HATU (33.4 mg, 0.088 mmol) and NMM (17.8 mg, 0.176 mmol). The reaction was left for 24 h. The DMF solution was then precipitated in cold diethyl ether and washed twice to obtain CP-His(Trt)-Boc (yield: 54.0 mg).

b. Synthesis of CP-His-NH_2

Removal of the protecting groups were achieved by adding a mixture of TFA 0.5 mL, TIPS 25 μL and DI water 25 μL to CP-His(Trt)-Boc (46.0 mg, 0.025 mmol) and stirring for 1 h. The resulting solution was then precipitated in cold diethyl ether and washed twice to give an off-white powder as CP-His-NH_2 (yield: 36.2 mg).

MS (ESI-ToF) (m/z): $[M+H]^+$ 1276.7 (calculated: 1276.7).

c. Synthesis of CP-His-PEG

CP-His-NH_2 (10.0 mg, 0.0078 mmol) and CH_3O-PEG-NHS (59.0 mg, 0.0112 mmol) were dissolved in 1.0 mL anhydrous DMF, with the addition of NMM (2.4 mg, 0.0235 mmol). The reaction was left for 24 h. Then the DMF solution was precipitated in cold diethyl ether. The precipitate was collected using centrifugation and dried under N_2. The resulting solid was then redissolved in 2 mL DCM and 10 mL diethyl ether was added dropwise to obtain precipitate. This process was repeated twice. Finally, CP-His-PEG was obtained by drying under vacuum as an off-white solid (yield: 30.0 mg).

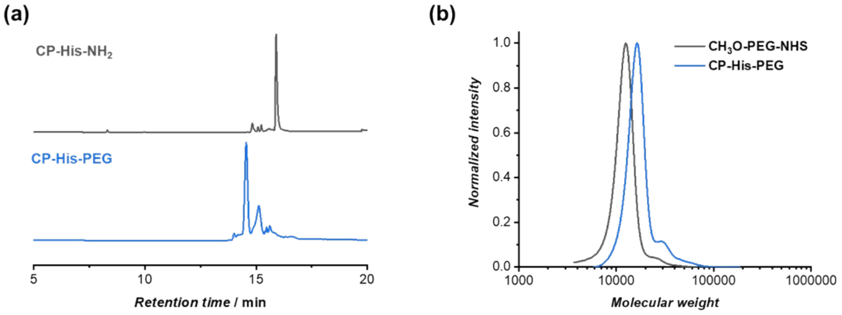

Figure S1 (a) HPLC spectra of CP-His-NH_2 and CP-His-PEG monitored by a UV detector at 280 nm; (b) GPC traces of CH_3O-PEG-NHS and CP-His-PEG.

3. Synthesis of CH_3O-PEG-BCN

CH_3O-PEG-BCN was synthesized according to procedures reported previously.[2]

4. Synthesis of His-CP-PEG

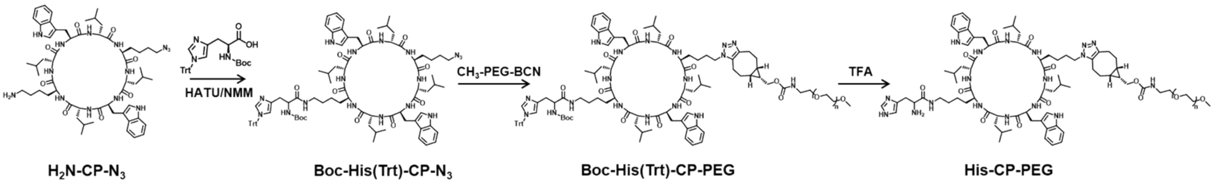

a. Synthesis of Boc-His(Trt)-CP-N_3

H_2N-CP-N_3 (20.0 mg, 0.018 mmol) and Boc-His(Trt)-OH (18.0 mg, 0.036 mmol) were dissolved in 1.5 mL anhydrous DMF, with the addition of HATU (13.7 mg, 0.036 mmol) and NMM (5.5 mg, 0.054 mmol). The reaction was left for 20 h. The DMF solution was then precipitated in cold diethyl ether and washed twice to obtain Boc-His(Trt)-CP-N_3 (yield: 22.0 mg).

MS (ESI-ToF) (m/z): $[M+H]^+$ 1586.9 (calculated: 1586.9).

b. Synthesis of Boc-His(Trt)-CP-PEG

Boc-His(Trt)-CP-N_3 (14.0 mg, 0.0088 mmol) and CH_3O-PEG-BCN (68.8 mg, 0.0132 mmol) were dissolved in 1.5 mL DMF. The reaction was left for 3 days. Then the DMF solution was precipitated in

cold diethyl ether. The precipitate was collected using centrifugation and dried under N_2. The resulting solid was then redissolved in 2 mL DCM and 10 mL diethyl ether was added dropwise to obtain precipitate. This process was repeated twice. Finally, Boc-His(Trt)-CP-PEG was obtained by drying under vacuum as an off-white solid (yield: 40.0 mg).

c. Synthesis of His-CP-PEG

Boc-His(Trt)-CP-PEG (35.0 mg) was dissolved in 0.5 mL DCM, with the addition of 0.5 mL TFA. The reaction was left for 3 h. Then the solution was precipitated in cold diethyl ether and washed twice. The obtained solid was dissolved in DI water and lyophilized to give an off-white powder as His-CP-PEG (yield: 28.0 mg).

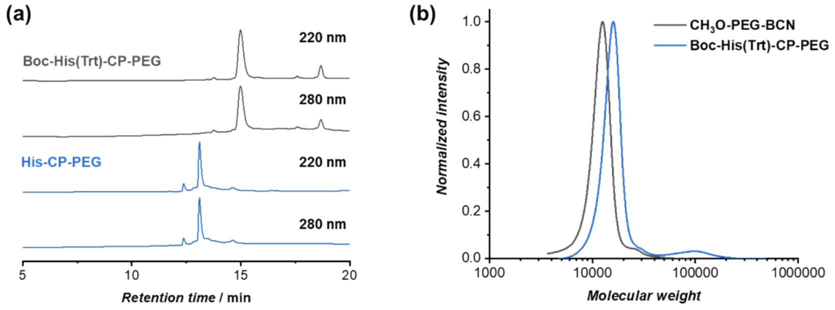

Figure S2 (a) HPLC spectra of Boc-His(Trt)-CP-PEG and His-CP-PEG monitored by a UV detector at 220 nm and 280 nm; (b) GPC traces of CH_3O-PEG-BCN and Boc-His(Trt)-CP-PEG.

5. Synthesis of His$_2$-CP-PEG

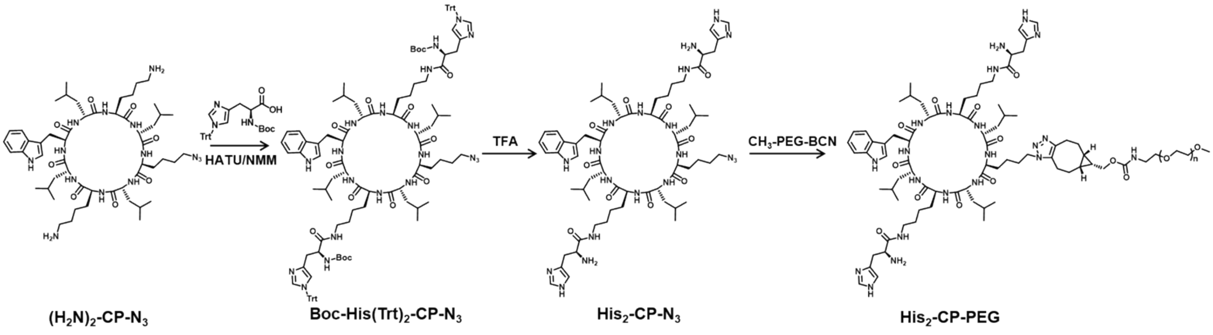

a. Synthesis of Boc-His(Trt)$_2$-CP-N_3

$(H_2N)_2$-CP-N_3 (20.0 mg, 0.019 mmol) and Boc-His(Trt)-OH (37.9 mg, 0.076 mmol) were dissolved in 2.0 mL anhydrous DMF, with the addition of HATU (29.0 mg, 0.076 mmol) and NMM (11.6 mg, 0.114 mmol). The reaction was left for 20 h. The DMF solution was then precipitated in cold diethyl ether and washed twice to obtain Boc-His(Trt)$_2$-CP-N_3 (yield: 26.0 mg).

b. Synthesis of His$_2$-CP-N$_3$

Removal of the protecting groups were achieved by adding a mixture of TFA 1 mL, TIPS 50 µL and DI water 50 µL to Boc-His(Trt)$_2$-CP-N$_3$ (24.0 mg, 0.012 mmol) and stirring for 3 h. The resulting solution was then precipitated in cold diethyl ether and washed twice to give an off-white powder as His$_2$-CP-N$_3$ (yield: 15.0 mg).

MS (ESI-ToF) (m/z): $[M+H]^+$ 1323.8 (calculated: 1323.8).

c. Synthesis of His$_2$-CP-PEG

His$_2$-CP-N$_3$ (10.0 mg, 0.0076 mmol) and CH$_3$O-PEG-BCN (59.0 mg, 0.0113 mmol) were dissolved in 1.5 mL DMF. The reaction was left for 3 days. Then the DMF solution was precipitated in cold diethyl ether. The precipitate was collected using centrifugation and dried under N_2. The resulting solid was then redissolved in 2 mL DCM and 10 mL diethyl ether was added dropwise to obtain precipitate. This process was repeated twice. The obtained solid was dissolved in DI water and lyophilized to give an off-white powder as His$_2$-CP-PEG (yield: 30.0 mg).

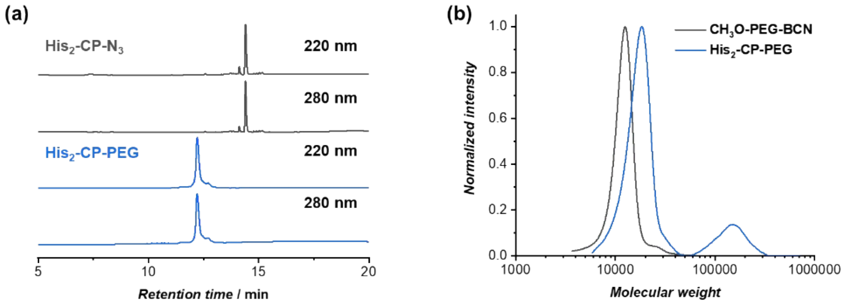

Figure S3 (a) HPLC spectra of His$_2$-CP-N$_3$ and His$_2$-CP-PEG monitored by a UV detector at 220 nm and 280 nm; (b) GPC traces of CH$_3$O-PEG-BCN and His$_2$-CP-PEG.

6. Synthesis of His-PEG

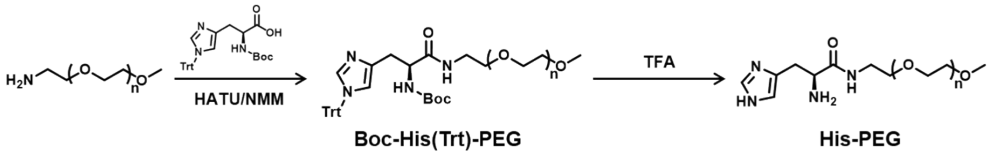

a. Synthesis of Boc-His(Trt)-PEG

CH$_3$O-PEG-NH$_2$ (100.0 mg, 0.02 mmol) and Boc-His(Trt)-OH (19.9 mg, 0.04 mmol) were dissolved in 1.5 mL anhydrous DMF, with the addition of HATU (15.2 mg, 0.04 mmol) and NMM (8.1 mg, 0.08 mmol). The reaction was left for 20 h and purified by precipitation in ice cold diethyl ether twice to give an off-white powder as the product Boc-His(Trt)-PEG (yield: 105 mg).

^1H NMR (400 MHz, DMSO-*d*): δ=7.84 (1H), 7.46 (9H), 7.12 (6H), 6.98 (1H), 4.23 (1H), 3.51 (PEG backbone), 3.24 (3H), 3.17 (2H), 2.81 (2H), 1.33 (9H).

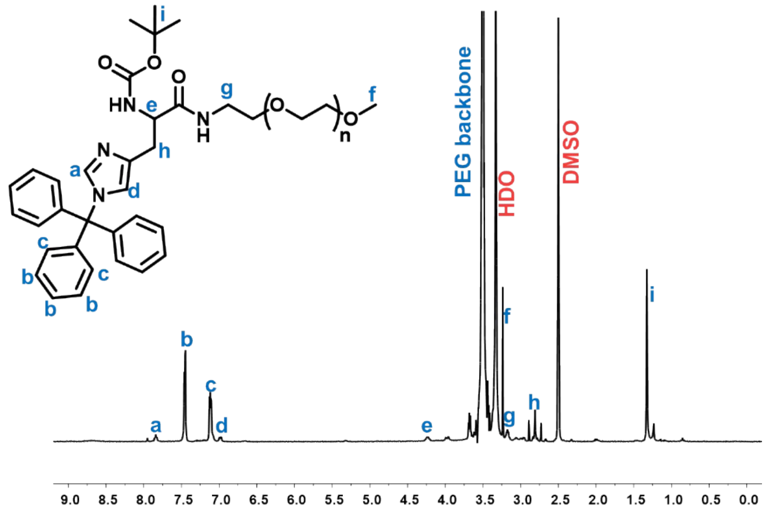

Figure S4 ^1H NMR spectrum of Boc-His(Trt)-PEG (400 MHz, DMSO-*d*).

b. Synthesis of His-PEG

Removal of the protecting groups was achieved by adding a mixture of trifluoroacetic acid (TFA, 0.5 mL), and DCM (1 mL) to Boc-His(Trt)-PEG (60 mg) and stirring for 2 hours. The resulting solution was then precipitated in ice cold diethyl ether and washed twice to give a white powder as the product His-PEG (yield: 45 mg).

^1H NMR (400 MHz, DMSO-*d*): δ=8.47 (1H), 7.43 (1H), 4.01 (1H), 3.50 (PEG backbone), 3.23 (3H), 3.12 (2H).

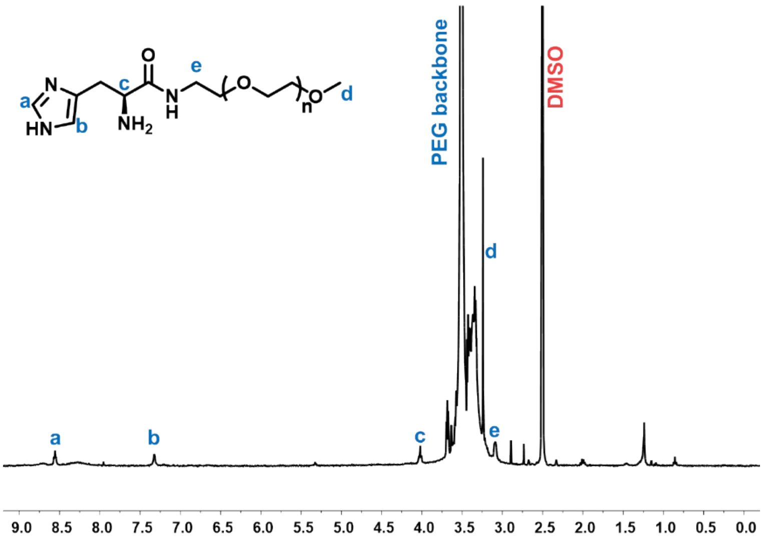

Figure S5 ^1H NMR spectrum of His-PEG (400 MHz, DMSO-*d*).

7. Synthesis of OCT-His-CP-PEG

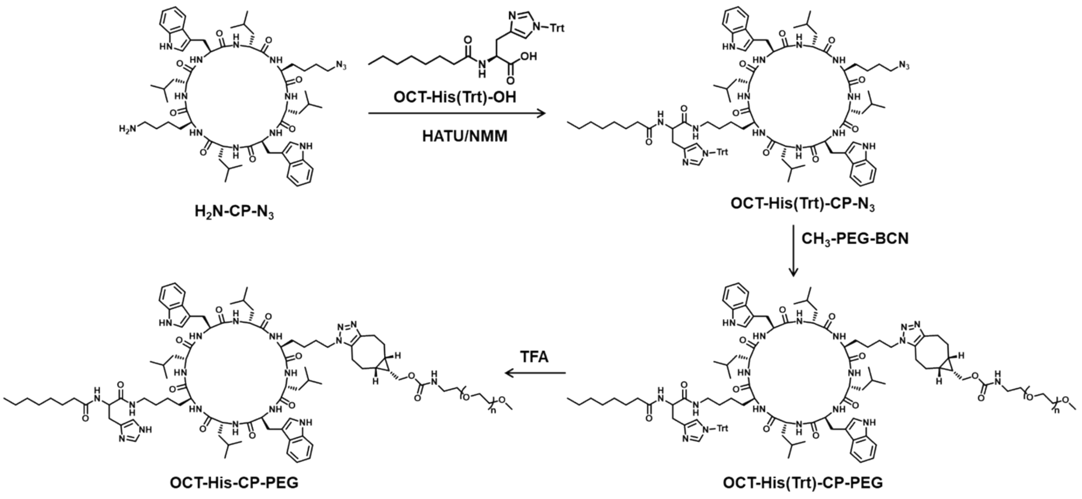

a. Synthesis of OCT-His(Trt)-OH

OCT-His(Trt)-OH was prepared *via* solid phase peptide synthesis (SPPS) on a Prelude Automated Peptide SynthesizerTM (Protein Technologies Inc.) using 2-chlorotrityl chloride resin as the solid support. Fmoc-His(Trt)-OH was coupled to the resin using DIPEA (4 eq.) in DMF, followed by capping of unreacted resin sites using a solution of MeOH:DIPEA:DCM (7:1:2, v/v/v). Deprotection of the Fmoc group of the amino acids was done using 20% piperidine in DMF. Subsequently octanoic acid was coupled under the condition of octanoic acid (5 eq.), HCTU (5 eq.) and NMM (10 eq.) in DMF. In the last step, OCT-His(Trt)-OH was cleaved from the resin by a solution of 20 vol % 1,1,1,3,3,3-hexafluoro-2-propanol (HFIP) in DCM. The solvent was evaporated to obtain OCT-His(Trt)-OH.

^{1}H NMR (400 MHz, DMSO-*d*): δ=7.94 (d, 1H), 7.38 (m, 9H), 7.29 (s, 1H), 7.06 (m, 6H), 6.67 (s, 1H), 4.37 (m, 1H), 2.89 (m, 1H), 2.73 (m, 1H), 2.0 (m, 2H), 1.39 (m, 2H), 1.20 (m, 8H), 0.83 (t, 3H).

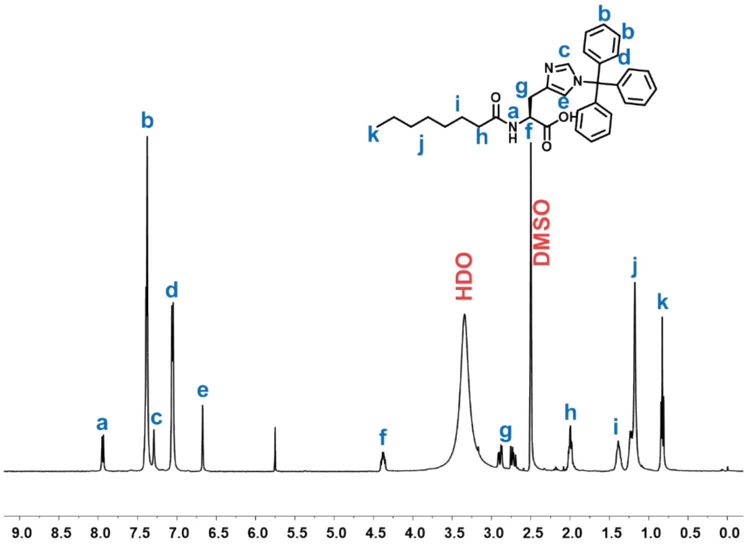

Figure S6 ^{1}H NMR spectrum of OCT-His(Trt)-OH (400 MHz, DMSO-*d*).

b. Synthesis of OCT-His(Trt)-CP-N_3

H_2N-CP-N_3 (15.0 mg, 0.014 mmol) and OCT-His(Trt)-OH (14.2 mg, 0.027 mmol) were dissolved in 1.5 mL anhydrous DMF, followed by the addition of HATU (10.3 mg, 0.027 mmol) and NMM (5.5 mg, 0.054 mmol). The reaction was left for 24 h. The DMF solution was then precipitated in cold diethyl ether and washed twice to obtain OCT-His(Trt)-CP-N_3 (yield: 24.0 mg).

MS (ESI-ToF) (m/z): $[M+H]^+$ 1612.9 (calculated: 1613.0).

c. Synthesis of OCT-His(Trt)-CP-PEG

OCT-His(Trt)-CP-N_3 (19.0 mg, 0.012 mmol) and CH_3O-PEG-BCN (91.9 mg, 0.018 mmol) were dissolved in 1.5 mL DMF. The reaction was left for 3 days. Then the DMF solution was precipitated in cold diethyl ether. The precipitate was collected using centrifugation and dried under N_2. The resulting solid was then redissolved in 2 mL DCM and 10 mL diethyl ether was added dropwise to obtain precipitate. This process was repeated twice. The obtained solid was dissolved in DI water and lyophilized to give an off-white powder as OCT-His(Trt)-CP-PEG (yield: 55.0 mg).

d. Synthesis of OCT-His-CP-PEG

OCT-His(Trt)-CP-PEG (40.0 mg) was dissolved in 0.5 mL DCM, with the addition of 0.5 mL TFA. The reaction was left for 3 h. Then the solution was precipitated in cold diethyl ether and washed twice. The obtained solid was dissolved in DI water and lyophilized to give an off-white powder as OCT-His-CP-PEG (yield: 35.0 mg).

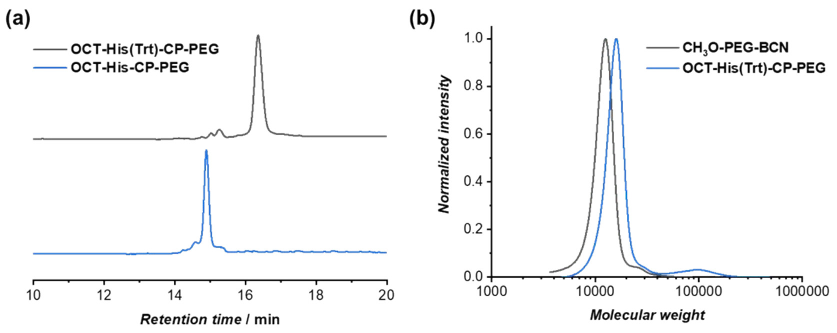

Figure S7 (a) HPLC spectra of OCT-His(Trt)-CP-PEG and OCT-His-CP-PEG monitored by a UV detector at 280 nm; (b) GPC traces of CH_3O-PEG-BCN and OCT-His(Trt)-CP-PEG.

S2. Self-assembling behaviour of CP-His-PEG, His-CP-PEG, His_2-CP-PEG, and OCT-His-CP-PEG

The self-assembly of the conjugates was realized simply by dissolving them into PBS buffer (pH=7.4) at different concentrations. For SAXS measurement, 5 mg mL^{-1} solutions were used. For SLS measurement, solutions of 3 different concentrations (1, 2, 3 mg mL^{-1}) were used.

SasView software was used to fit the SAXS data, using a core-shell cylinder model. SLD values were calculated based on the molecular structure of the conjugate and solvent. The radius of the core value was fixed at 5 Å, representing the radius of the cyclic peptide itself.

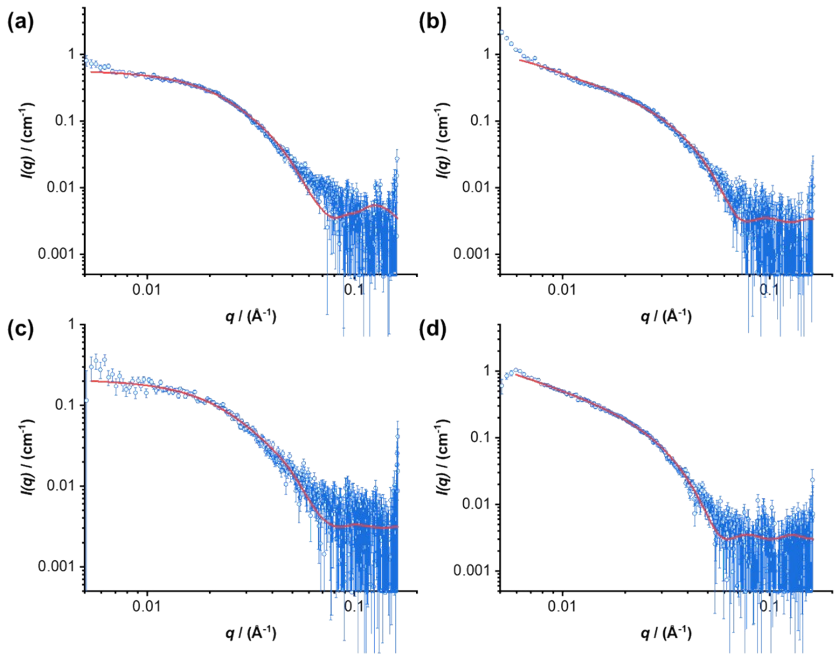

Figure S8 SAXS scattering data of (a) CP-His-PEG (b) His-CP-PEG (c) His2-CP-PEG (d) OCT-His-CP-PEG, and fitting to a core–shell cylinder model.

Table S1 Fitting parameters using a core–shell cylinder model by SasView software.

	**CP-His-PEG**	**His-CP-PEG**	**His_2-CP-PEG**	**OCT-His-CP-PEG**
**Scale**	0.494±0.023	0.0079±0.0059	0.0033±0.0008	0.0076±0.0050
**Background**	0.001*	0.002*	0.002*	0.003*
**SLD_{core}/ × 10^{-6} $Å^{-2}$**	12.53*	12.57*	12.63*	12.57*
**SLD_{shell}/ × 10^{-6} $Å^{-2}$**	9.441±0.002**	9.896±0.205**	9.969±0.732**	9.788±0.142**
**$SLD_{solvent}$/ × 10^{-6} $Å^{-2}$**	9.39*	9.39*	9.39*	9.39*
**$Radius_{core}$/ Å**	5*	5*	5*	5*
**$Thickness_{shell}$/Å**	57.7±0.3	47.3±0.7	44.5±1.5	60.2±0.8
**Length/Å**	127±3	571±36	139±6	2000*

* The parameters are fixed.

** SLD_{shell} is allowed to vary during the fitting considering the solvation effect.

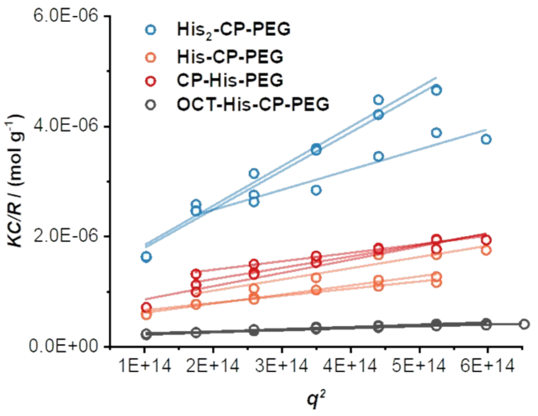

Figure S9 SLS data of CP-His-PEG, His-CP-PEG, His_2-CP-PEG and OCT-His-CP-PEG measured in water.

Table S2 N_{agg} values of CP-His-PEG, His-CP-PEG, His_2-CP-PEG and OCT-His-CP-PEG obtained from SLS data.

	**CP-His-PEG**	**His-CP-PEG**	**His$_2$-CP-PEG**	**OCT-His-CP-PEG**
MW_{unimer} / (g mol^{-1})	6390	6496	6575	6622
dn/dc	0.14	0.14	0.14	0.14
$MW_{aggregate}$ / (g mol^{-1})	(1.16±0.17) × 10^6	(1.93±0.24) × 10^6	(7.95±1.57) × 10^5	(5.11±0.34) × 10^6
N_{agg}	181±27	297±37	121±24	772±52

S3. Determination of the catalytic activity towards PNPA or PNPB hydrolysis

a. Determination of the extinction coefficient of 4-nitrophenol in PBS buffer

A series of the hydrolysis product 4-nitrophenol solutions in PBS buffer (pH=7.4) with different concentrations were prepared (25, 50, 100, 250, 500 μM). The UV-vis spectra were then measured using a quartz cuvette with a path length of 2 mm. After plotting the absorbance at 400 nm against 4-nitrophenol concentration, a linear fitting was carried out to obtain the slope value as (0.00208±0.00003) μM^{-1}. The extinction coefficient of 4-nitrophenol at 400 nm in PBS buffer was calculated according to Beer-Lambert law, $A = \varepsilon c l$, which gives a value of 10400 M^{-1} cm^{-1}.

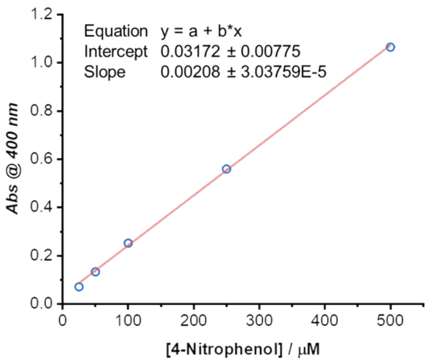

Figure S10 Plot of absorbance at 400 nm *vs.* 4-nitrophenol concentration in PBS buffer.

b. Kinetics experiment

The catalytic activities of the histidine-containing compounds were determined using either 4-nitrophenyl acetate (PNPA) or 4-nitrophenyl butyrate (PNPB) as the substrate. The hydrolytic product is 4-nitrophenol with an absorption band peaked at 400 nm. The reaction rate was determined by monitoring the change of absorbance at 400 nm over time, which was subsequently converted into concentration using the measured extinction coefficient of 4-nitrophenol. Stock solutions of the histidine-containing compounds were prepared in PBS buffer, and the stock solution of PNPA or PNPB was prepared in DMSO. PNPA or PNPB hydrolysis was carried out in a quartz cuvette containing a certain amount of catalyst and 1 mM PNPA or PNPB with a total volume of 500 μL at a controlled temperature.

The initial hydrolytic rates were calculated by a linear fitting of the evolution of absorbance at 400 nm over reaction time at the very beginning of the reaction, assuming the substrate concentration held approximately constant.

c. Determination of apparent activation energy (E_a)

The hydrolysis of PNPA or PNPB was monitored at different temperatures (25, 40, 60, and 80 °C) at His-CP-PEG concentrations of 0, 40, and 80 μM, respectively, while PNPA or PNPB concentration was kept at 1 mM. The initial hydrolytic rates were calculated by a linear fitting of the evolution of absorbance at 400 nm over reaction time at the very beginning of the reaction, assuming the substrate concentration held approximately constant. By fitting the evolution of initial hydrolytic rates as a function of reaction temperatures using Arrhenius equation, E_a related to the PNPA or PNPB hydrolysis could be obtained.

$v_0 = Ae^{\frac{-Ea}{RT}}$, which could be transformed to $ln(v_0) = lnA - E_a/RT$

A linear fitting is conducted between $ln(v_0)$ and $1/T$, which gives the slope as $-E_a/R$.

Table S3 E_a values obtained by fitting the evolution of initial hydrolytic rates as a function of reaction temperatures using Arrhenius equation.

Substrate	PNPA			PNPB		
[His-CP-PEG]	0	40 µM	80 µM	0	40 µM	80 µM
Slope	-8388±228	-6911±87	-5915±177	-9217±187	-5372±136	-4830±173
E_a / (kJ mol^{-1})	69.7±1.9	57.5±0.7	49.2±1.5	76.6±1.6	44.7±1.1	40.2±1.4
R^2	0.998	0.999	0.997	0.999	0.998	0.996

Table S4 Summary of the catalytic performance of self-assembling peptide amphiphiles or amphiphilic short peptides.

	Catalyst	Assembly	Condition	Substrate	k_{cat} (s^{-1})	K_m (mM)	k_{cat}/K_m (M^{-1} s^{-1})	Ref
1	Peptide amphiphile	Nanofiber	HEPES 50 mM, pH=7.4, 25 °C	DNPA	1.67×10^{-2}	0.845	19.76	4
2	CoA-HSD	Nanofiber	HEPES 10 mM, pH=7.5 35 °C	PNPA	2×10^{-3}	16.29	0.186	5
3	Azo-GFGH	Nanofiber	PBS, pH=7.4, 25 °C	PNPA	3.7×10^{-3}	15.56	0.233	6
4	AM1	Nanofiber	PBS 50 mM, pH=7.46, 27 °C	PNPA	1.27×10^{-3}	1.43	0.89	7
5	Fe-AM1	Metallo-hydrogel		PNPA	2.82×10^{-2}	45.12	0.63	
6	AM1	Nanofiber		PNPB	7.1×10^{-4}	0.75	0.95	
7	Fe-AM1	Metallo-hydrogel		PNPB	3.0×10^{-3}	4.07	0.75	
6	PepNTs-His-Arg	Nanotube	HEPES 10 mM, pH 7.5, 25 °C	PNPA	1.38×10^{-3}	0.76	1.82	8
7	Hydrogelator	Hydrogel	pH=7	PNPA	2.1×10^{-2}	4.0	5.25	9
8	Peptide triad	Nanofiber	PBS, pH=7.4, 25 °C	PNPA	4.41×10^{-3}	0.035	126.62	10
9	HGC/RGC peptides	Vesicle	PB, pH=8, 37 °C	PNPA	1.14×10^{-2}	5.65	2.02	11
10	Q11HR$_{max}$	Nanofiber	PBS, pH=7.4	PNPA	2.64×10^{-3}	17.63	0.15	12
11	R8	Modified resin	Tris, pH=8.9	PNPB	2.72×10^{-2}	12	2.268	13
12	His-CP-PEG	Polymeric nanotubes	PBS 10 mM, pH=7.4, 25 °C	PNPA	1.07×10^{-2}	11.3	0.947	This work

DNPA: 2,4-dinitrophenyl acetate

PNPA: 4-nitrophenyl acetate

PNPB: 4-nitrophenyl butyrate

1. E. D. H. Mansfield, M. Hartlieb, S. Catrouillet, J. Y. Rho, S. C. Larnaudie, S. E. Rogers, J. Sanchis, J. C. Brendel and S. Perrier, *Soft Matter*, **2018**, *14*, 6320-6326.

2. Q. Song, S. Goia, J. Yang, S. C. L. Hall, M. Staniforth, V. G. Stavros and S. Perrier, *J. Am. Chem. Soc.*, **2021**, *143*, 382-389.

3. Q. Song, J. Yang, J. Y. Rho and S. Perrier, *Chem. Commun.,* **2019**, *55*, 5291-5294.

4. M. O. Guler and S. I. Stupp, *J. Am. Chem. Soc.*, **2007**, *129*, 12082-12083.

5. M. Wang, Y. Lv, X. Liu, W. Qi, R. Su and Z. He, *ACS Appl. Mater. Interfaces*, **2016**, *8*, 14133-14141.

6. Y. Zhao, B. Lei, M. Wang, S. Wu, W. Qi, R. Su and Z. He, *J. Mater. Chem. B*, **2018**, *6*, 2444-2449.

7. K. Gayen, K. Basu, D. Bairagi, V. Castelletto, I. W. Hamley and A. Banerjee, *ACS Appl. Bio Mater.*, **2018**, *1*, 1717-1724.

8. Z. Huang, S. Guan, Y. Wang, G. Shi, L. Cao, Y. Gao, Z. Dong, J. Xu, Q. Luo and J. Liu, *J. Mater. Chem. B*, **2013**, *1*, 2297-2304.

9. N. Singh, M. P. Conte, R. V. Ulijn, J. F. Miravet and B. Escuder, *Chem. Commun.*, **2015**, *51*, 13213-13216.

10. G. Gulseren, M. A. Khalily, A. B. Tekinay and M. O. Guler, *J. Mater. Chem. B*, **2016**, *4*, 4605-4611.

11. N. Ma, F. Li, S. Li, S. Chu, L. Han, S. Liu, T. Yan, R. Tian, Q. Luo and J. Liu, *Nanoscale*, **2019**, *11*, 3521-3526.

12. C. Zhang, X. Xue, Q. Luo, Y. Li, K. Yang, X. Zhuang, Y. Jiang, J. Zhang, J. Liu, G. Zou and X.-J. Liang, *ACS Nano*, **2014**, *8*, 11715-11723.

13. M. D. Nothling, A. Ganesan, K. Condic-Jurkic, E. Pressly, A. Davalos, M. R. Gotrik, Z. Xiao, E. Khoshdel, C. J. Hawker, M. L. O'Mara, M. L. Coote and L. A. Connal, *Chem*, **2017**, *2*, 732-745.
